# AI-based chest CT quantification of interstitial lung disease in idiopathic inflammatory myopathies: agreement with expert visual assessment in 107 patients

**DOI:** 10.1186/s12890-026-04201-6

**Published:** 2026-02-26

**Authors:** Youlia Kuzmanovic, Amira Benattia, Amandine Laporte, Kubéraka Mariampillai, Yves Allenbach, Yurdagül Uzunhan, Pierre-Yves Brillet, Phillipe A. Grenier, Victoria Donciu, Nicoletta Pasi, Olivier Benveniste, Alban Redheuil, Samia Boussouar

**Affiliations:** 1 Sorbonne Université, Assistance Publique-Hôpitaux de Paris, Département d’Imagerie Cardiovasculaire et Thoracique (ICT), Hôpital Pitié-Salpêtrière, 47-83 Boulevard de l’Hôpital, Paris, 75013 France; 2 Assistance Publique-Hôpitaux de Paris, Hôpital Saint-Louis, Service de Pneumologie, Paris, France; 3 Sorbonne Université, Laboratoire d’Imagerie Biomédicale, Inserm, CNRS, Paris, France; 4 Assistance Publique-Hôpitaux de Paris, Hôpital Cochin, Service de Radiologie ostéo-articulaire, Paris, France; 5 Département d’Oncologie, Agence Nationale de Sécurité du Médicament et des produits de santé ANSM, Saint-Denis, France; 6 Sorbonne Université, Assistance Publique-Hôpitaux de Paris, Département de médecine interne et immunologie clinique, Hôpital Pitié-Salpêtrière, Paris, France; 7 Université Sorbonne Paris Nord, Assistance Publique-Hôpitaux de Paris, Hôpital Avicenne, Service de Pneumologie, Bobigny, France; 8 Université Sorbonne Paris Nord, Assistance Publique-Hôpitaux de Paris, Hôpital Avicenne, Service de Radiologie, Bobigny, France; 9 Direction de la Recherche clinique et de l’Innovation, Hôpital Foch, Suresnes, France

**Keywords:** Artificial intelligence, Interstitial lung disease, Idiopathic inflammatory myopathies, Computed tomography

## Abstract

**Background:**

Interstitial lung disease (ILD) is a determinant of morbidity and mortality in idiopathic inflammatory myopathies (IIM), but chest HRCT evaluation remains observer-dependent. Artificial intelligence (AI) may provide reproducible quantitative assessment. We compared AI-based quantification of ILD with expert visual scoring in IIM.

**Methods:**

In this monocentric retrospective study, 107 patients with IIM-associated ILD from a national myositis registry were included. One representative chest HRCT per patient was evaluated by a thoracic radiologist using a semi-quantitative lobar score and by a commercially available AI tool for lung texture analysis. AI-derived volumes were converted to the same 5-point scale as the visual score. Correlations were assessed with Spearman coefficients and agreement with Cohen’s kappa.

**Results:**

All CTs were suitable for visual assessment and 106/107 (99%) for AI analysis. AI identified ground-glass opacities (GGO) as the predominant abnormality, with a lower-lobe predominance. Correlations between AI and radiologist scores were strong for normal lung (*r* = 0.77) and moderate for GGO (*r* = 0.64) and consolidation (*r* = 0.60), but weaker for reticulations (*r* = 0.34) and honeycombing (*r* = 0.42). Agreement was good for GGO (κ = 0.70) and consolidation (κ = 0.60), moderate for reticulations (κ = 0.37) and low for honeycombing (κ = 0.16).

**Conclusion:**

In IIM-associated ILD, AI-based chest HRCT quantification showed good agreement with expert visual assessment, particularly for GGO and consolidation, but was less reliable for complex fibrotic patterns. AI may support more objective and reproducible evaluation of interstitial involvement, as a complement to expert interpretation.

## Introduction

Idiopathic inflammatory myopathies (IIMs) are a heterogeneous group of systemic connective tissue diseases characterised by autoimmune inflammatory muscle involvement and a broad spectrum of extra-muscular manifestations. Among these, interstitial lung disease (ILD) is one of the most frequent and severe complications, affecting up to 40% of patients and significantly worsening prognosis [1–3]. In some IIM subsets, ILD may even dominate the clinical picture and drive outcomes more than muscle involvement itself.

High-resolution computed tomography (HRCT) plays a central role in the detection and characterisation of ILD in IIM. Typical patterns include non-specific interstitial pneumonia (NSIP), organising pneumonia (OP) or mixed NSIP/OP forms, which differ from the usual interstitial pneumonia (UIP) pattern classically seen in idiopathic pulmonary fibrosis [4–6]. The extent and distribution of parenchymal abnormalities provide important prognostic information and influence therapeutic decisions, including the initiation or escalation of immunosuppressive or antifibrotic therapy [7–9]. However, visual HRCT assessment remains largely semi-quantitative and is subject to inter- and intra-observer variability, particularly for subtle ground-glass opacities (GGOs), fine reticulations and early fibrotic changes, and may be difficult to standardise in multicentre settings [10–12].

Over the past decade, computer-based quantitative imaging and artificial intelligence (AI) algorithms have emerged as promising tools to provide objective and reproducible assessment of ILD on chest HRCT [13–15]. Automated lung segmentation and texture analysis can quantify the volume and spatial distribution of normal lung, GGO, consolidation, reticulation, honeycombing and emphysema, and quantitative HRCT metrics have been associated with disease severity, functional impairment and outcomes in fibrotic ILDs, suggesting a potential role as imaging biomarkers. Nevertheless, data on AI-based HRCT analysis specifically in IIM-associated ILD are scarce, and the distinctive patterns in this setting – including the frequent overlap of inflammatory and fibrotic lesions and the relatively low prevalence of IIM-ILD – may challenge the performance of tools trained mainly on other ILD populations [14–16].

Therefore, the aim of this study was to compare a commercially available AI-based chest HRCT analysis tool with semi-quantitative visual scoring by an experienced thoracic radiologist for the quantification of ILD lesions in patients with IIM-associated ILD.

## Materials and methods

### Population

This monocentric retrospective observational study included patients enrolled in the Myositis Registry of a national reference centre for neuromuscular diseases. Inclusion criteria were: (i) confirmed diagnosis of idiopathic inflammatory myopathy according to the 2017 ACR/EULAR criteria [17]; (ii) available myositis-specific antibody (MSA) results when performed as part of routine care; and (iii) at least one available chest HRCT examination, where ILD was identified. IIM subtypes were assigned based on routine clinico-serological diagnosis: anti-synthetase syndrome required anti–aminoacyl-tRNA synthetase (anti-ARS) positivity with a compatible clinical phenotype, and immune-mediated necrotizing myopathy was classified according to standard clinico-serological criteria (including anti-SRP when present). Part of this registry-based cohort has been previously reported [5, 18] MSAs were assessed in our center using commercial line-blot immunoassays (Euroline Myositis 4G, Euroimmun, Lübeck, Germany, since May 2014); pre-2014 testing procedures are detailed in Laporte et al. [5].Muscle biopsy was not a predefined inclusion criterion for this imaging substudy and was not systematically collected for the present analysis; classification relied on the 2017 ACR/EULAR criteria, with biopsy performed only when clinically indicated as part of routine care. Institutional Review Board approval was obtained (Comité de Protection des Personnes Ile-de-France VI, Groupe Hospitalier Pitié-Salpêtrière). All patients gave written informed consent for inclusion in the registry and use of anonymised data.

### Chest imaging

**Fig. 1:**
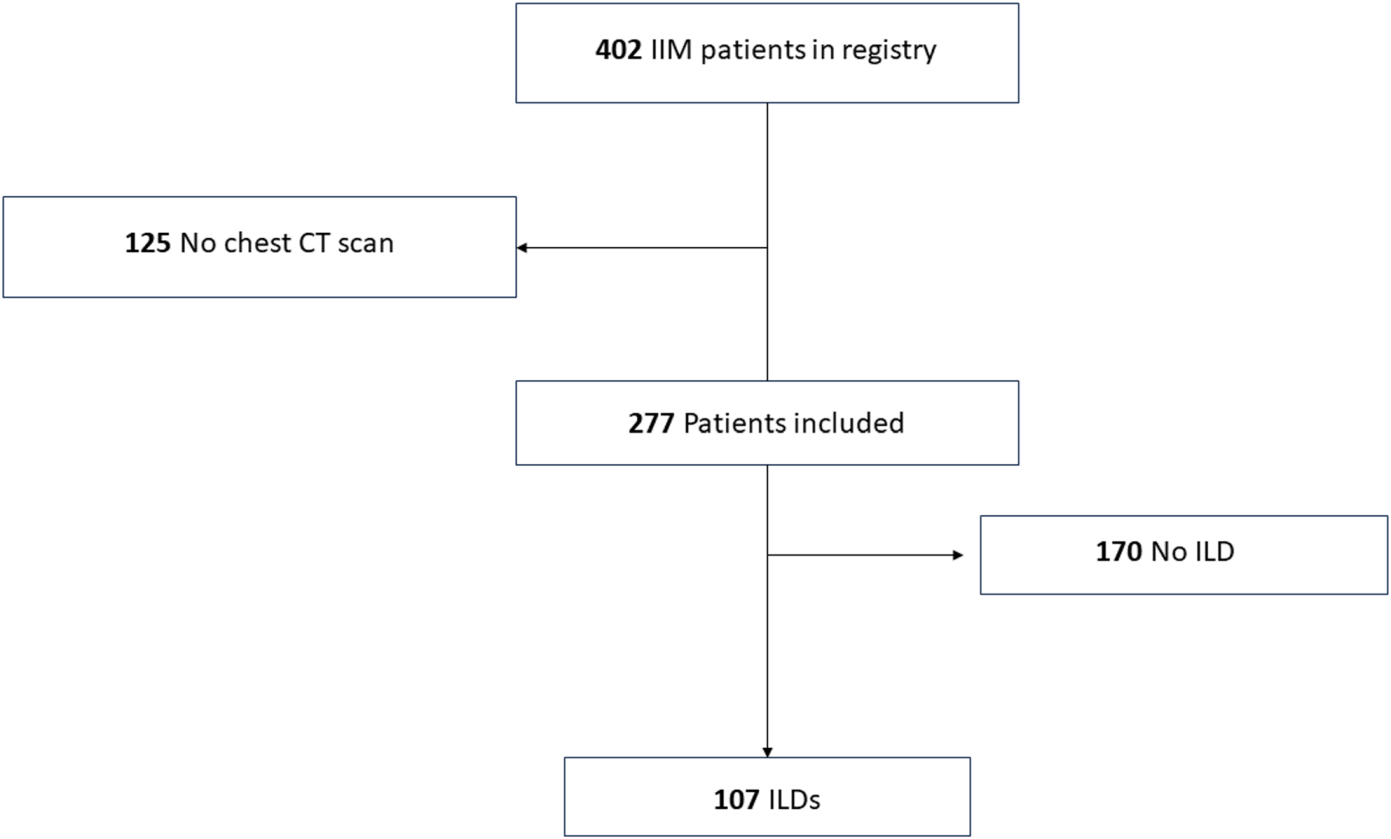
Study flowchart

Among the 402 patients initially enrolled, 277 underwent chest HRCT (study flowchart, Fig. 1) which constitutes the imaging-available subset used for the present analyses. HRCT examinations were performed using multidetector scanners from different manufacturers (Siemens Healthineers, GE Healthcare, Philips Medical Systems). All scans were acquired in full inspiration, in the supine position, without intravenous contrast. Prone acquisitions were added in 18 patients. Images were reconstructed using high-spatial-frequency algorithms with slice thickness ranging from 0.6 mm to 1.5 mm.

### HRCT analysis

All chest HRCT examinations were reviewed within the registry workflow by thoracic radiologists (PG, 30 years’ experience; SB, 10 years; and AL, junior reader) and classified as either normal (strictly normal or showing only non-ILD findings) or abnormal with ILD-related lesions (e.g., ground-glass opacities, reticulation, traction bronchiectasis and/or honeycombing), based on contemporary international criteria [6, 7, 19]. Disagreements were resolved by consensus. For the AI agreement analysis, semi-quantitative visual scoring was performed by a dedicated reader (YK) blinded to clinical data and to AI outputs.

In patients with multiple HRCTs during follow-up, one examination was selected per patient during a clinically stable period, defined as stable ILD extent and pattern, and excluding scans performedduring acute exacerbations or intercurrent events (e.g. infection, heart failure or drug toxicity). This was done to minimise confounding by transient acute changes and to ensure that quantitative measures primarily reflected chronic ILD involvement.

Each selected CT examination was independently analysed twice: once by a radiologist and once by a commercially available AI-based software (AVIEW Lung Texture v.1.1.44.27-win, Coreline, Seoul, Republic of Korea), which applies deep learning for lung texture analysis.

For each exam, elementary pulmonary lesions (ground-glass opacities, reticulations, consolidation, honeycombing, emphysema) and normal lung parenchyma were identified. The extent of each finding was assessed globally and at the lobar level, using a 5-point semi-quantitative visual scoring system (0: none; 1: 1–24%; 2: 25–49%; 3: 50–74%; 4: 75–100%) [20].

AI-derived results were expressed as absolute lesion volumes and percentages of total lung volume for each feature. For comparative analysis, percentages were converted into the same 5-point scale used for visual scoring.

### Statistics

Quantitative variables are summarized as median [interquartile range (IQR)] unless stated otherwise. Categorical variables are reported as absolute frequencies and corresponding percentages. The association between AI-derived continuous metrics and radiologists’ semi-quantitative scores was assessed using Spearman’s rank correlation coefficient (ρ). Correlation strength was interpreted as follows: weak (ρ < 0.40), moderate (ρ = 0.40–0.59), strong (ρ = 0.60–0.79), very strong (ρ ≥ 0.80). Agreement between categorical variables was evaluated using Cohen’s kappa statistic (κ), with the following interpretation: slight (κ = 0.00–0.20), fair (κ = 0.21–0.40), moderate (κ = 0.41–0.60), substantial (κ = 0.61–0.80), almost perfect (κ = 0.81–1.00). All statistical tests were two-tailed, with significance defined as *p* < 0.05.

## Results

### Study population

Of the 277 patients with at least one chest HRCT available, 170 had no evidence of ILD and 107 had HRCT-confirmed ILD and were included in the present analysis Patient selection is summarized in Fig. 1. Among 107 included patients with IIM-associated ILD, 74 were female (69%) and the median age was 53 years [IQR 41–66] (Table 1). Anti-Jo1 was the most prevalent antibody (38.3%), followed by anti-SRP (12.1%), anti-MDA5 (10.3%) and others. The most common IIM subtype was anti-synthetase syndrome (48.6%) (Table 1). The predominant HRCT ILD patterns were NSIP (60/107, 56.1%), NSIP/OP (41/107, 38.3%), and UIP (6/107, 5.6%).

**Table 1:** Baseline characteristics of the 107 patients with idiopathic inflammatory myopathy–associated interstitial lung disease

Characteristics	*n*	%
Patients - Total	107	100
Age, years, median [IQR]	53 [41; 66]	
Sex		
Female	74	69.2
Male	33	30.8
Myositis-specific antibodies (MSAs)		
Known status	102	95.3
MSA-positive	80	74.7
Anti-Jo1	41	38.3
Anti-SRP	13	12.1
Anti-MDA5	11	10.3
Anti-PL12	8	7.5
Anti-PL7	3	2.8
Anti-cN1a	2	1.9
Anti-TIF1	1	0.9
Anti-SAE	1	0.9
Seronegative	22	20.6
Unknown status	5	4.7
IIM Subtype		
Anti-synthetase syndrome	52	48.6
Dermatomyositis	17	15.9
Polymyositis	17	15.9
Immune-mediated necrotizing myopathy	15	14.0
Inclusion body myositis	6	5.6

Data are presented as n (%) or median [interquartile range]

*MSA* Myositis-specific antibody, *SRP* Signal recognition particle, *MDA5* Melanoma differentiation-associated gene 5, *PL* Anti-aminoacyl-tRNA synthetase antibodies, *cN1a* Cytosolic 5’-nucleotidase 1 A, *TIF1* Transcription intermediary factor 1, *SAE* Small ubiquitin-like modifier activating enzyme

### AI-based quantification

Only lung parenchyma reconstructions were analysed. One case (0.9%) presented a partial segmentation failure of the left lung, and was excluded from volumetric analysis. No other technical failure occurred.

Automated analysis was performed without user interaction and was typically completed within a few minutes per scan.

Quantified lesions were generally limited in extent. Ground-glass opacities were the most frequent and extensive abnormality, with a median involvement of 4.7% (IQR, 1.5–13.4%) of the total lung volume, predominantly in the lower lobes (left lower lobe: 7.6%; right lower lobe: 6.9%). Consolidation (median: 0.4%), reticulations (1.6%), and minimal volumes of honeycombing and emphysema (both < 0.1%) were also detected (Table 2).

**Table 2:** AI-based quantitative CT analysis of lung lesions in patients with idiopathic inflammatory myopathy–associated interstitial lung disease

Variables	Mean or median	SD or IQR
Total lung volume (ml)	3392	1257
Normal lung (%)	82.4	18.4
Consolidation		
Total lung (%)	0.4	[0.1 ; 1.0]
RUL (%)	0.1	[0.0 ; 1.0]
ML (%)	0.1	[0.0 ; 0.3]
RLL (%)	0.4	[0.1 ; 1.6]
LUL (%)	0.2	[0.0 ; 0.5]
LLL (%)	0.5	[0.1 ; 1.7]
Ground-glass opacities		
Total lung (%)	4.7	[1.5 ; 13.4]
RUL (%)	0.6	[0.1 ; 3.5]
ML (%)	1.1	[0.2 ; 7.4]
RLL (%)	7.6	[2.3 ; 17.3]
LUL (%)	0.7	[0.2 ; 5.7]
LLL (%)	6.9	[2.0 ; 21.1]
Reticulations		
Total lung (%)	1.6	[0.4 ; 5.8]
RUL (%)	0.3	[0.0 ; 1.5]
ML (%)	1.4	[0.1 ; 2.1]
RLL (%)	2.5	[0.5 ; 8.0]
LUL (%)	0.4	[0.1 ; 1.9]
LLL (%)	2.5	[0.5 ; 10.1]
Honeycombing		
Total lung (%)	0.0	[0.0 ; 0.1]
RUL (%)	0.0	[0.0 ; 0.0]
ML (%)	0.0	[0.0 ; 0.0]
RLL (%)	0.1	[0.0 ; 0.1]
LUL (%)	0.0	[0.0 ; 0.0]
LLL (%)	0.1	[0.0 ; 0.1]
Emphysema		
Total lung (%)	0.0	[0.0 ; 0.1]
RUL (%)	0.0	[0.0 ; 0.1]
ML (%)	0.0	[0.0 ; 0.0]
RLL (%)	0.0	[0.0 ; 0.1]
LUL (%)	0.0	[0.0 ; 0.1]
LLL (%)	0.0	[0.0 ; 0.1]

Data are presented as mean ± standard deviation or median [interquartile range], as appropriate. Values represent the percentage of total lung or lobar volume

*RUL* Right upper lobe, *ML* Middle lobe, *RLL* Right lower lobe, *LUL* Left upper lobe, *LLL* Left lower lobe, *GGO* Ground-glass opacity

### AI vs. radiologist: correlation and agreement

Spearman correlations between AI-derived percentages and radiologist semi-quantitative scores were strong for normal lung parenchyma (ρ = 0.77) and moderate for GGO (ρ = 0.64) and consolidation (ρ = 0.60) (Table 3). Correlation was weaker for reticulation (ρ = 0.34), honeycombing (ρ = 0.42) and emphysema (ρ = 0.24).

**Table 3:** Correlation between AI-derived lesion extent and semi-quantitative radiologist scores

Variables	Spearman’s ρ	*p*-value (*p*)
Total normal lung parenchyma	0.77	< 0.001
Consolidation	0.60	< 0.001
Ground-glass opacities	0.64	< 0.001
Reticulations	0.34	
Honeycombing	0.42	< 0.001
Emphysema	0.24	0.011

Spearman correlation coefficients (ρ) and p-values for normal lung and each lesion type

*GGO* Ground-glass opacity

On visual scoring, the proportion of patients with each abnormality was as follows: ground-glass opacities in 93/107 (86.9%), reticulation in 64/107 (59.8%), consolidation in 36/107 (33.6%), honeycombing in 8/107 (7.5%), and emphysema in 5/107 (4.7%). Extent categories are detailed in Table 4.

**Table 4:** Semi-quantitative categories of lesion extent according to AI and radiologist, and agreement between methods

Variables	AI	Radiologist	Cohen’s Kappa	95% CI	
Normal lung (%)			0.39	0.30–0.47	
0–1	0	0			
1–24	1 (0.9)	1 (0.9)			
25–49	8 (7.5)	11 (10.3)			
50–74	21 (19.6)	18 (16.8)			
75–100	78 (72.9)	77 (72)			
Consolidation (%)			0.60	0.45–0.76	
0–1	70 (73.8)	71 (66.4)			
1– 24	28 (26.2)	35 (32.7)			
25–49	0	1 (0.9)			
50–74	0	0			
75–100	0	0			
Ground-glass opacities (%)			0.70	0.58–0.82	
0–1	21 (19.6)	14 (13.1)			
1–24	71 (66.4)	78 (72.9)			
25–49	12 (11.2)	10 (9.3)			
50–74	3 (2.8)	4 (3.7)			
75–100	0	1 (0.9)			
Reticulations (%)				0.37	0.20–0.53
0–1	44 (41.1)	43 (40.2)			
1–24	62 (57.9)	63 (58.9)			
25–49	1 (0.9)	1 (0.9)			
50–74	0	0			
75–100	0	0			
Honeycombing				0.16	0.04–0.29
0–1	96 (89.7)	99 (92.5)			
1–24	9 (8.4)	5 (4.7)			
25–49	2 (1.9)	3 (2.8)			
50–74	0	0			
75–100	0	0			
Emphysema				0.51	0.20–0.82
0–1	100 (93.5)	102 (95.3)			
1–24	7 (6.5)	4 (3.7)			
25–49	0	2 (1.9)			
50–74	0	0			
75–100	0	0			

Values are n (%). Extent categories represent the percentage of total lung volume involved (0; 1–24; 25–49; 50–74; 75–100). Cohen’s κ and 95% confidence intervals (CI) are shown for each lesion type

*GGO* Ground-glass opacity

When comparing semi-quantitative categories, agreement was good for GGO (κ = 0.70), moderate for consolidation (κ = 0.60), reticulation (κ = 0.37), and emphysema (κ = 0.51) and low for honeycombing (κ = 0.16) (Table 4).

**Fig. 2:**
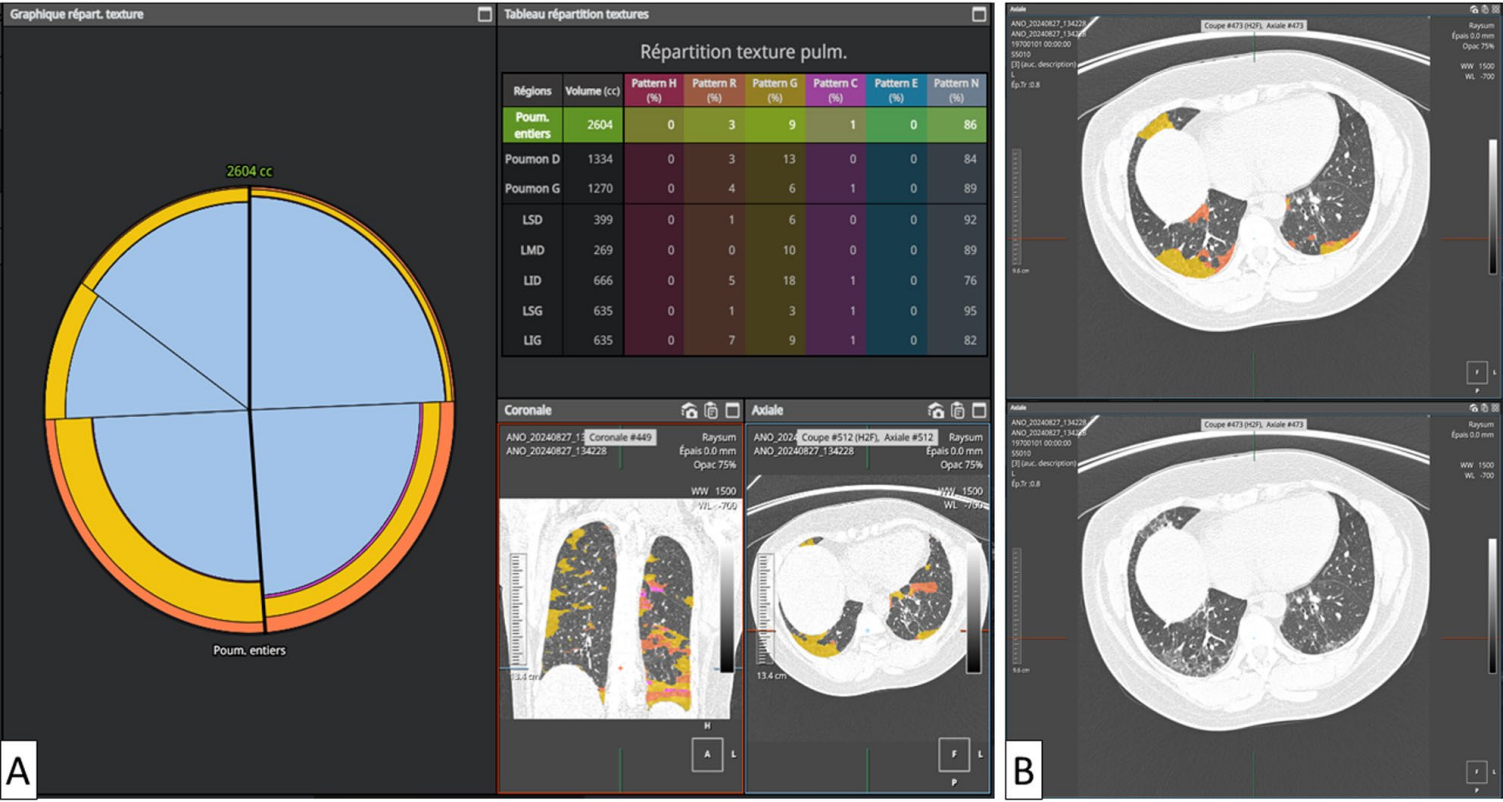
Lung texture analysis in a 47-year-old woman with anti-Jo1–positive idiopathic inflammatory myopathy–associated interstitial lung disease (IIM-ILD). Axial high-resolution CT (HRCT) images are shown at two adjacent basal levels (lower lobes); the patient’s right side is displayed on the left. **A** Quantitative report summarising the volumetric extent of each texture class, expressed as a percentage of total lung volume and for each lobe. Texture classes are labelled as follows: N (normal lung), G (ground-glass opacities), C (consolidation), R (reticulation), H (honeycombing), and E (emphysema). **B** Axial HRCT image showing diffuse, predominantly lower-lobe ground-glass opacities with the AI-generated colour-coded texture overlay (top) and the corresponding original HRCT image (bottom), consistent with a predominantly inflammatory, non-fibrotic ILD pattern that may inform immunosuppressive management

**Fig. 3:**
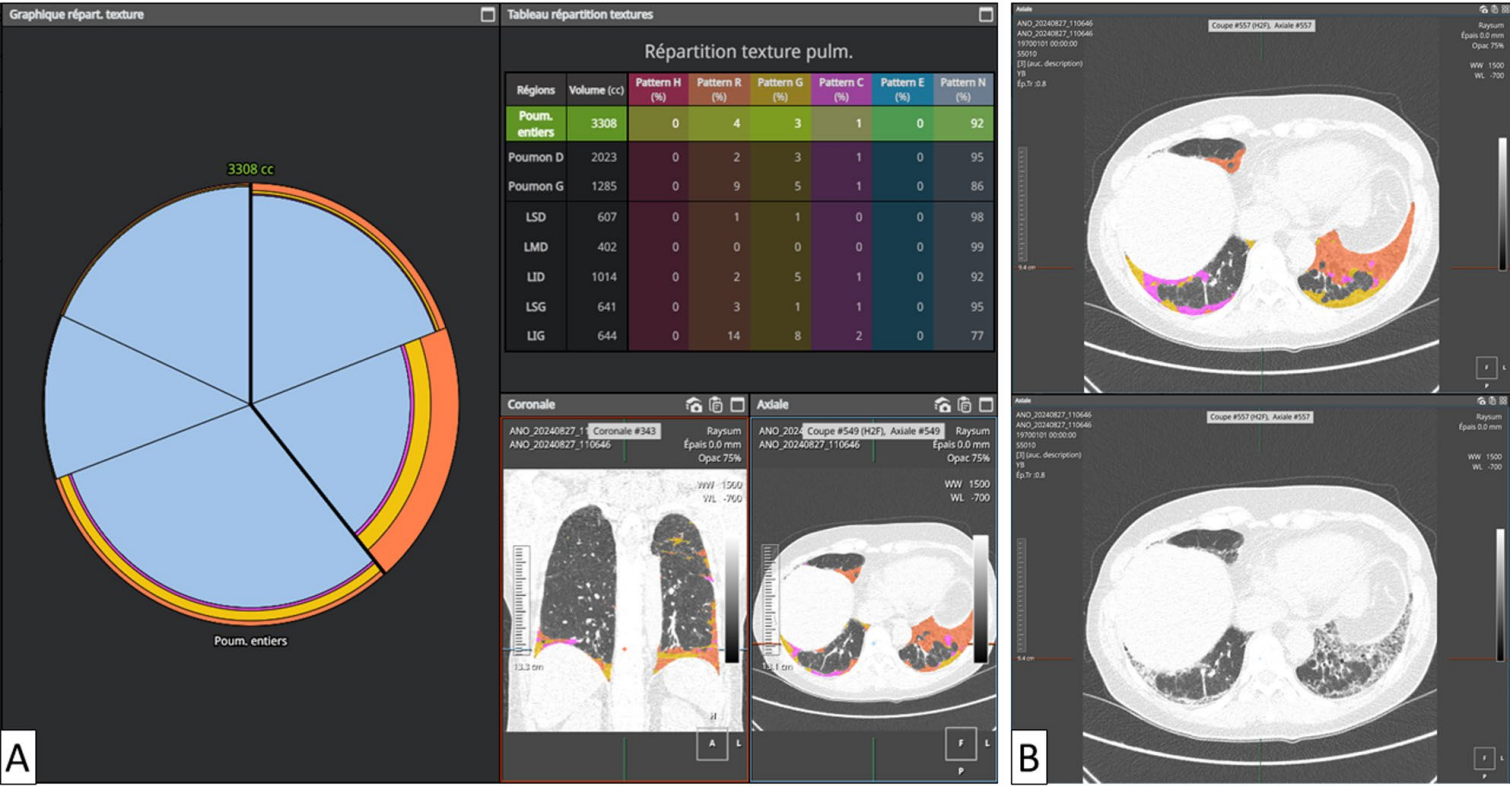
Lung texture analysis in a 36-year-old woman with anti-Jo1–positive idiopathic inflammatory myopathy–associated ILD. Axial images are shown at the level of the lung bases (lower lobes); the patient’s right is on the left side of the image. Panels A and B show the same axial slice. **A** Quantitative report summarising the volumetric extent of each texture class as a percentage of total lung volume and of each lobe. Texture classes are labelled as follows: N (normal lung), G (ground-glass opacities), C (consolidation), R (reticulation), H (honeycombing), and E (emphysema). **B** Corresponding HRCT images, with AI-based segmentation and colour-coded overlay of texture classes (top) and the original HRCT image (bottom). This case illustrates heterogeneous ILD involvement with both ground-glass opacities and fibrotic-related abnormalities and exemplifies how quantitative outputs may support longitudinal monitoring

Figures 2 and 3 illustrate typical lower-lobe–predominant IIM-associated ILD patterns, with either purely inflammatory GGO or mixed GGO–fibrotic disease, and show how AI can separately quantify these components.

### Lobar analysis

Most lesions showed a basal predominance, except emphysema, which was more apical. GGO in lower lobes showed moderate agreement between AI and radiologist (κ = 0.43–0.52), while reticulations had poor agreement at the lobar level (κ < 0.10) (Table 5).

**Table 5:** Lobar analysis of correlation and agreement between AI and radiologist for each lesion type

Variables	Spearman’s ρ (*p*-value)	(*p*)	Cohen’s Kappa	95% CI
Consolidation				
RLL	0.55	< 0.001	0.19	0.12–0.27
LLL	0.49	< 0.001	0.19	0.07–0.31
GGO				
RLL	0.60	< 0.001	0.43	0.32–0.55
LLL	0.58	< 0.001	0.52	0.39–0.64
Reticulations				
RLL	0.14	0.156	0.03	-0.03-0.10
LLL	0.22	0.022	0.05	-0.01-0.12
Honeycombing				
RLL	0.38	< 0.001	0.26	0.08–0.44
LLL	0.41	< 0.001	0.27	0.06–0.48
Emphysema				
RUL	0.35	< 0.001	0.06	0.00-0.13
LUL	0.25	0.011	0.04	-0.02-0.10

Spearman correlation coefficients (ρ) and Cohen’s κ (95% CI) are shown for lower lobes (right lower lobe, RLL; left lower lobe, LLL) for consolidation, GGO, reticulations and honeycombing, and for upper lobes (right upper lobe, RUL; left upper lobe, LUL) for emphysema

*GGO* Ground-glass opacity

## Discussion

In this monocentric cohort of 107 patients with IIM-associated ILD, we compared an AI-based quantitative chest HRCT analysis tool with semi-quantitative visual scoring by an experienced thoracic radiologist. AI-based analysis was feasible in almost all patients and consistently quantified GGO as the predominant abnormality, with a typical lower-lobe predominance. Overall, there was strong agreement between AI and the radiologist for normal lung and moderate agreement for GGO and consolidation, while concordance was weaker for reticulations, honeycombing and emphysema. These findings support the role of AI as a complementary tool to expert visual assessment for the objective quantification of ILD in IIM, particularly for inflammatory and non-fibrotic lesions.

The distribution and nature of HRCT lesions in our cohort are consistent with the characteristic radiological patterns described in IIM-ILD, where NSIP, OP or mixed NSIP/OP patterns predominate and classic UIP is relatively uncommon [4–6, 21]. Similar patterns have been reported in myositis-associated ILD and anti-synthetase syndrome, as well as in broader reviews of IIM-ILD and connective tissue disease-associated ILD [8, 21–23]. Our findings are in line with those of Li et al., who reported NSIP and OP patterns in 67% and 26% of cases, respectively, in a large cohort of IIM patients with ILD [9]. As expected, GGO (86.9%) and reticulations (59.8%) were the most frequent elementary lesions, and abnormalities were mainly basal and subpleural, as typically described in IIM-ILD and other CTD-related ILDs [5, 21, 23, 24]. This concordance with previous radiological series supports the representativeness of our population. By quantifying lesion volumes, the AI tool confirmed the predominance of GGO and lower-lobe involvement, suggesting that it can capture the main topographic and morphologic features of IIM-ILD.

Radiologist-based semi-quantitative assessment revealed limited pulmonary involvement in most patients, with interstitial abnormalities affecting less than 25% of total lung volume and 75–100% of lung parenchyma preserved in 72% of cases. This relatively mild extent of disease contrasts with the more diffuse and fibrotic patterns typically seen in idiopathic pulmonary fibrosis [7, 23]. From a clinical perspective, this reflects a population in whom accurate quantification of inflammatory lesions is crucial for guiding immunosuppressive treatment and monitoring disease progression, as ILD is a major determinant of prognosis in IIM [1–4, 15, 21].

Our correlation results show strong alignment between AI and radiologist scores for normal lung (*r* = 0.77) and moderate alignment for GGO (*r* = 0.64) and consolidation (*r* = 0.60), consistent with a good concordance for the most prevalent and/or extensive parenchymal components in this cohort. Agreement was strongest for GGO in the lower lobes, which are classically involved in CTD-related ILD, including IIM-associated ILD [4, 5, 21, 23]. We observed strong correlations between AI-derived and reader-derived measures, alongside only moderate Cohen’s kappa for some features (e.g., normal lung). This reflects that Spearman’s correlation captures whether measurements co-vary across subjects (i.e., whether patients are similarly ranked), whereas kappa assesses exact agreement after discretisation into ordinal categories. Here, AI-derived continuous percentages and semi-quantitative visual scores generally varied in parallel, but small differences around category cut-offs (often a one-category shift) reduced categorical agreement, resulting in moderate kappa despite high correlation. Clinically, this is relevant because these components often reflect active inflammatory involvement and are commonly monitored over time to inform management. As this analysis was restricted to HRCT-confirmed IIM-ILD, the serological distribution may not reflect the full IIM spectrum and some MSA subsets (e.g., anti-Mi2) may be under-represented.

In contrast, agreement between AI and the radiologist was weaker for reticulations (*r* = 0.34) and honeycombing (*r* = 0.42). Several factors likely contribute to this discrepancy. First, reticulation and early fibrosis often overlap with GGO in NSIP, making it challenging even for expert readers to delineate clear boundaries between inflammatory and fibrotic components [5, 6, 21]. Some AI tools, including the one used in our study, treat GGO and reticulations as mutually exclusive categories, whereas they are frequently intertwined and spatially overlapping in IIM-ILD, which may lead to differences in labelling. Second, the textural signatures of reticulation and honeycombing may be underrepresented or heterogeneous in the training datasets of commercial AI tools that were primarily developed and validated for other ILDs, such as idiopathic pulmonary fibrosis [10, 14, 15, 25]. Third, detection of honeycombing is known to be problematic for both humans and machines. Watadani et al. reported only moderate interobserver agreement for honeycombing (κ = 0.40–0.58), largely due to confusion with mimickers such as traction bronchiectasis or emphysema [10], a diagnostic challenge that automated algorithms are also likely to face. Our results indicate that AI agreement was higher for inflammatory abnormalities than for honeycombing/emphysema in this cohort. Given the scarcity of these end-stage features and the limited disease spectrum, the corresponding findings should be interpreted cautiously and warrant validation in cohorts with more advanced fibrotic disease. Importantly, fibrotic involvement in IIM-associated ILD is often reflected by reticulation and traction bronchiectasis rather than honeycombing.We also observed limited agreement for emphysema quantification (*r* = 0.24). This may reflect the relatively low burden of emphysema in this specific population, potential threshold-related misclassification of low-attenuation areas, and the difficulty of separating emphysema from cystic ILD lesions on purely density-based analysis [14, 23]. In addition, AI may underperform in detecting subtle or upper-lobe–dominant abnormalities, where visual pattern recognition and anatomical context remain crucial. For pneumologists, these findings underline that AI outputs regarding emphysema and advanced fibrosis should still be interpreted with caution and always integrated with expert visual review and clinical data.

When comparing semi-quantitative categories, agreement was good for GGO (κ = 0.70) and consolidation (κ = 0.60), and moderate for reticulations (κ = 0.37). The lowest agreement was observed for honeycombing (κ = 0.16), underscoring the difficulty of reliably identifying infrequent and morphologically complex patterns. For normal lung, kappa agreement was only moderate (κ = 0.39), which may partly reflect a tendency for radiologists to visually overestimate minimal involvement once a lesion is perceived. While AI provides continuous, volume-based estimates and may classify some borderline areas as normal, the human eye tends to attribute a small but non-zero extent of disease whenever an abnormality is seen [11, 14, 25, 26]. This illustrates both the potential added precision of AI for minimal disease and the need to interpret automated results in light of visual assessment.

Our study has several limitations that should be acknowledged. Its retrospective, single-centre design and relatively limited sample size may restrict the generalisability of the findings, although IIM-ILD remains a rare condition and our cohort is among the larger series with standardised CT analysis [4, 5, 18, 21]. The visual reference standard relied on a single reader, interobserver variability was not assessed and no formal inter-reader agreement metrics were calculated, so performance may differ in less specialised settings [10–12]. HRCT scans were acquired on multiple scanners with non-standardised acquisition parameters, which may introduce variability in quantitative measurements. Previous work using the same software reported a variability of approximately 1% in fibrosis quantification between repeated acquisitions on the same day, with higher variability when reconstruction parameters differ substantially [27].We evaluated only one commercially available AI software, whose training data and internal algorithms are not fully transparent. Performance may vary across different tools, and our results cannot be extrapolated to other systems without further validation [13–16, 25, 28].

We did not formally analyse associations between AI-derived metrics and clinical outcomes such as pulmonary function, survival or treatment response, which would be an important next step, particularly given the emerging role of quantitative HRCT biomarkers in prognostication and disease monitoring in ILD [3, 13–15]. Pulmonary function tests (FVC and DLCO) were not available systematically and were not consistently temporally matched to the index HRCT, precluding robust structure–function analyses and the assessment of HRCT–PFT discordance. A standardised disease-duration measure could not be derived reliably because symptom onset/diagnosis dates were not uniformly recorded and CT timing varied across patients.

Several limitations relate to cohort characteristics and lesion distribution. ILD was predominantly mild, and the extent distribution was highly skewed toward low categories (0–1% and 1–24%), which may affect agreement estimates—particularly categorical metrics—through class imbalance and floor effects; results should therefore be interpreted cautiously in low-extent categories. Honeycombing and emphysema were scarce, with very low median volumes. In the context of IIM-associated ILD, predominantly NSIP; NSIP/OP in our population, this mainly reflects limited representation of end-stage UIP-type changes; accordingly, assessment of AI performance for honeycombing/emphysema is constrained by lesion scarcity and floor effects, and very small non-zero volumes should be interpreted cautiously. In addition, clinical ILD status (symptoms, whether ILD was clinically recognised at baseline, and ILD-directed treatment) was not systematically collected in this retrospective imaging substudy; therefore, the present findings should be interpreted as applying primarily to HRCT-established ILD rather than clinically overt ILD. This study was designed as an agreement analysis in HRCT-confirmed IIM-ILD and not as a diagnostic accuracy study for ILD detection or pattern classification. Finally, although AI-based quantification may provide standardised, less reader-dependent measurements, reproducibility was not assessed because only one CT per patient was analysed; dedicated repeat-scan studies are needed to quantify test–retest, inter-scan, and interobserver reproducibility of AI-derived measures.Despite these limitations, our results provide clinically meaningful information for pneumologists. They suggest that AI-based chest HRCT analysis is feasible in IIM-ILD and can reliably quantify normal lung and inflammatory lesions, particularly GGO, even in a population with predominantly mild and lower-lobe–predominant disease. At the same time, they underscore the current limitations of AI for complex fibrotic patterns such as reticulation and honeycombing and emphasise the need for continued expert radiological input within a multidisciplinary framework, integrating radiological, clinical and functional data, especially when decisions about prognosis and long-term management are being made [6, 11, 21, 25].

In conclusion, AI-based quantitative HRCT analysis shows promising concordance with expert visual assessment for the evaluation of ILD in patients with idiopathic inflammatory myopathies, particularly with respect to GGO and consolidation. These tools may support more objective and reproducible assessment of lung involvement and could be integrated into routine practice and clinical trials as a complement to, rather than a replacement for, expert human interpretation. Future multicentre, prospective studies incorporating longitudinal imaging, pulmonary function data and clinical outcomes are warranted to better define the prognostic value and utility of AI-derived imaging biomarkers in IIM-associated ILD and their role in monitoring response to immunosuppressive or antifibrotic therapies.

## Data Availability

The datasets generated and/or analysed during the current study are not publicly available due to institutional policies and French data protection regulations regarding patient confidentiality, and cannot be shared.

## Abbreviations

AI: Artificial intelligence
ATS: American Thoracic Society
CI: Confidence interval
CPP: Comité de Protection des Personnes
CT: Computed tomography
CTD: Connective tissue disease
ERS: European Respiratory Society
EULAR: European Alliance of Associations for Rheumatology
GE: General Electric
GGO: Ground-glass opacity
HRCT: High-resolution computed tomography
IIM: Idiopathic inflammatory myopathy
ILD: Interstitial lung disease
IQR: Interquartile range
IRB: Institutional Review Board
κ: Cohen’s kappa coefficient
LLL: Left lower lobe
LUL: Left upper lobe
ML: Middle lobe
MSA: Myositis-specific antibody
NSIP: Non-specific interstitial pneumonia
OP: Organising pneumonia
ρ: Spearman’s rank correlation coefficient
RLL: Right lower lobe
RUL: Right upper lobe
SD: Standard deviation
UIP: Usual interstitial pneumonia
